# Serological and histopathological assessment of galactose-deficient immunoglobulin A1 deposition in kidney allografts: A multicenter prospective observational study

**DOI:** 10.1371/journal.pone.0281945

**Published:** 2023-02-16

**Authors:** Tadashi Sofue, Hideyo Oguchi, Masahiko Yazawa, Makoto Tsujita, Kenta Futamura, Morikuni Nishihira, Mariko Toyoda, Toshiki Kano, Hitoshi Suzuki

**Affiliations:** 1 Department of Cardiorenal and Cerebrovascular Medicine, Kagawa University, Kagawa, Japan; 2 Department of Nephrology, Toho University Faculty of Medicine, Tokyo, Japan; 3 Division of Nephrology and Hypertension, Department of Internal Medicine, St. Marianna University School of Medicine, Kawasaki, Japan; 4 Department of Nephrology, Masuko Memorial Hospital, Nagoya, Japan; 5 Department of Kidney Disease Center, Japanese Red Cross Aichi Medical Center, Nagoya Daini Hospital, Aichi, Japan; 6 Department of Nephrology, Yuuai Medical Center, Okinawa, Japan; 7 Department of Nephrology, Japanese Red Cross Kumamoto Hospital, Kumamoto, Japan; 8 Department of Nephrology, Juntendo University Faculty of Medicine, Tokyo, Japan; Universidade de Sao Paulo, BRAZIL

## Abstract

**Background:**

Recurrent immunoglobulin A (IgA) nephropathy is an important risk factor for kidney allograft loss. However, there is no classification system for IgA deposition in kidney allografts based on serological and histopathological evaluation of galactose-deficient IgA1 (Gd-IgA1). This study aimed to establish a classification system for IgA deposition in kidney allografts based on serological and histological evaluation of Gd-IgA1.

**Methods:**

This multicenter prospective study included 106 adult kidney transplant recipients in whom an allograft biopsy was performed. Serum and urinary Gd-IgA1 levels were investigated in 46 transplant recipients who were IgA-positive and classified into four subgroups according to the presence or absence of mesangial Gd-IgA1 (KM55 antibody) deposits and C3.

**Results:**

Minor histological changes without an acute lesion were observed in recipients with IgA deposition. Fourteen (30%) of the 46 IgA-positive recipients were KM55-positive and 18 (39%) were C3-positive. The C3 positivity rate was higher in the KM55-positive group. Serum and urinary Gd-IgA1 levels were significantly higher in KM55-positive/C3-positive recipients than in the other three groups with IgA deposition. Disappearance of IgA deposits was confirmed in 10 of 15 IgA-positive recipients in whom a further allograft biopsy was performed. The serum Gd-IgA1 level at the time of enrollment was significantly higher in recipients in whom IgA deposition continued than in those in whom it disappeared (*p* = 0.02).

**Conclusions:**

The population with IgA deposition after kidney transplantation is serologically and pathologically heterogeneous. Serological and histological assessment of Gd-IgA1 is useful for identifying cases that should be carefully observed.

## Introduction

Kidney transplantation is preferred over dialysis for patients with end-stage renal disease because it results in significantly better quality of life, longer patient survival, lower cardiovascular risk, and less medical expenditure [1–3]. Recurrent immunoglobulin A (IgA) nephropathy (IgAN) is an important risk factor for allograft loss [4], and half of all allograft losses in recipients with IgAN as the original disease are due to recurrence [5]. Recurrent glomerulonephritis can be detected by both protocol and episode allograft biopsies [6]. Although there has been a report of an increase in the recurrence rate at 10 years after transplantation [5], this finding was based on episode biopsies. Asymptomatic IgA deposition detected by protocol biopsy performed within a few years after transplantation has been found to have a favorable prognosis [7, 8].

While some cases of recurrent asymptomatic IgA deposition in allografts detected by protocol biopsy progress to symptomatic IgAN, asymptomatic IgA deposition in donated kidneys that appears pathologically identical to that in allografts disappears rapidly after transplantation [9–14]. IgA deposition in allograft kidneys is considered to be heterogeneous and can be classified into several groups based on pathological features, serum biomarkers, and abnormalities in urine [15]. IgA deposition without abnormal urinalysis and IgAN with abnormal urinalysis should be considered separately [16]. However, previous studies have been unable to distinguish between cases of asymptomatic IgA deposition in allografts that progress to IgAN with abnormal urinalysis and those that do not. Therefore, even if IgA deposition is found in allograft kidneys, the need for treatment cannot be determined.

Galactose-deficient immunoglobulin A1 (Gd-IgA1) has been identified to be a critical mechanism in the pathogenesis of IgAN [17]. It has been discussed whether Gd-IgA1 (KM55) staining can distinguish primary IgAN from secondary IgA deposition in the native kidney [18–23]. KM55 staining has also been found to be positive in allograft kidneys with IgAN [24, 25]. It has also been reported that serum and urinary Gd-IgA1 quantification is useful for evaluation of the activity of IgAN in the native kidney [18, 26, 27], and the recurrence of IgAN in allograft kidneys is associated with a high Gd-IgA1 levels [28–30]. However, until now, there has been no system for classification of IgA deposition in allografts that includes both serological and histopathological assessment of Gd-IgA1.

The aim of this multicenter prospective observational study was to predict disease activity and prognosis for IgA deposition in allograft kidneys based on serological and histological assessment of Gd-IgA1.

## Materials and methods

### Subjects

We prospectively investigated 174 adult kidney transplant recipients who underwent protocol or episode allograft kidney biopsies between June 2016 and October 2020. Participants were recruited to the study from June 2016 to October 2020. Recipients who were at least 1-year post-transplant were included. Both deceased and living donor kidney transplants were included. Protocol or episode allograft kidney biopsies were performed at all participating institutions according to the indications in the clinical practice guidelines [31].

Recipients with IgA deposition in the allograft biopsy at the time of enrollment were investigated to determine whether there had been any further allograft biopsies performed within the subsequent 3 years; if so, these biopsies were checked for IgA deposits.

Informed consent was obtained from all study participants. The protocols, patient information, and informed consent forms were reviewed and approved by the Ethics Committee of Kagawa University (#H27-228) and each participating facility. The study is registered in the UMIN Clinical Trials Registry (UMIN000021922). The authors had access to information that could identify individual participants during or after data collection at their institution but could not access to information that could identify individual participants at other institutions.

### Biopsy and pathological analyses

All allograft biopsies were performed using a 16-G or 18-G biopsy needle at each participating hospital. The biopsy specimens were fixed in formalin and embedded in paraffin. A portion of the biopsy tissue was reserved for frozen sections. The paraffin sections were stained with hematoxylin and eosin or Masson trichrome. A biopsy was deemed adequate if it contained at least eight glomeruli. Sclerotic glomeruli (%) was defined as the percentage of sclerotic glomeruli in the total number of glomeruli counted [32].

IgA, Gd-IgA1, and C3 deposition in the allograft biopsy specimens was examined using immunofluorescence staining, as described in a previous study [24]. Unstained paraffin sections with a thickness of 4 μm were prepared at each facility and sent to the central evaluation facility at Juntendo University for immunostaining and evaluation of IgA and Gd-IgA1. The primary antibody used for Gd-IgA1 immunostaining was KM55 (#10777; Immuno-Biological Laboratories, Gunma, Japan). Immunostaining for C3 and C4d was performed using frozen sections at each institution. Pathological diagnosis was made by a pathologist at each institution who was not otherwise involved in the study and who was blinded to the subgroup classification based on Gd-IgA1 staining. Positive immunostaining was defined as +1 or more. IgA staining was classified as 1+ or 2+. No 3+ cases were observed.

IgA deposition was defined as positive mesangial IgA deposition. Electron microscopy results were available for 37 of the 46 recipients with positive mesangial IgA deposition and confirmed dense paramesangial deposits. Mesangial expansion was defined as hypercellularity of mesangial cells (i.e., >5 mesangial cells/mesangial area), and expansion of mesangial matrices in at least one glomerulus [33]. Banff score, T-cell-mediated rejection, and antibody-mediated rejection were defined based on The Banff 2019 Kidney Meeting Report [34].

### Study design

**Fig 1:**
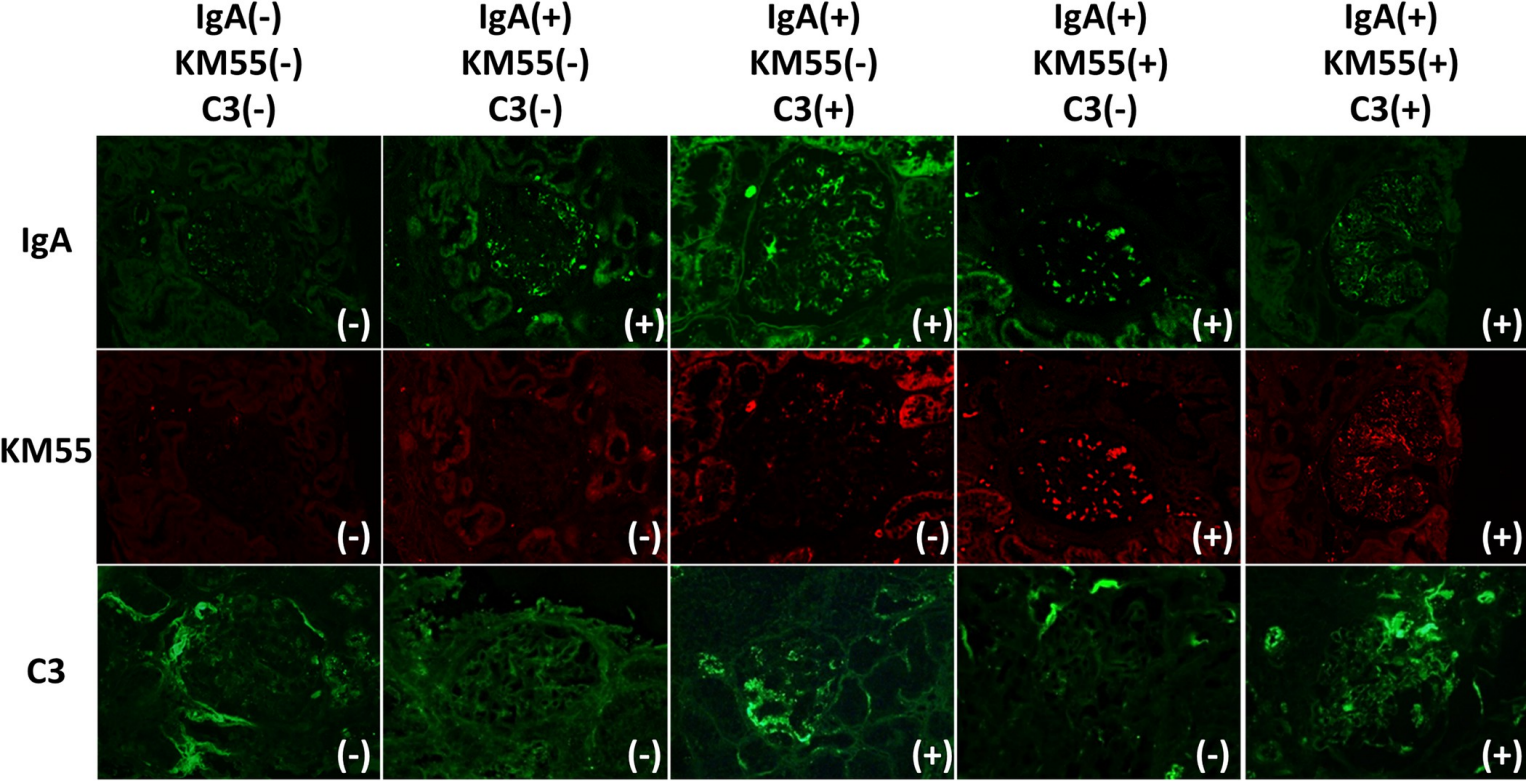
Immunostaifning characteristics in the subgroups. IgA, immunoglobulin A; IgA(−), IgA-negative group; IgA(+), IgA-positive group; KM55(−), KM55-negative group; KM55(+), KM55-positive group; KM55(−)C3(−), KM55-negative/C3-negative group; KM55(−)C3(+), KM55-negative/C3-positive group; KM55(+)C3(−), KM55-positive/C3-negative group; KM55(+)C3(+), KM55-positive/C3-positive group.

A flow chart summarizing the patient classification process is shown in S1 Fig. Ten of the 174 recipients were excluded because of a lack of blood samples (n = 8) or an absence of pathological data (n = 2). The remaining 164 recipients with adequate data were classified into two subgroups according to whether mesangial IgA deposition was absent (IgA-negative recipients, n = 113) or present (IgA-positive recipients, n = 46). Sixty of the 113 IgA-negative recipients were randomly extracted and confirmed to be representative of the original group (S1 Table). Finally, the study included 106 recipients. The 46 IgA-positive recipients were classified into four subgroups according to mesangial KM55 and C3 deposition status, as follows: KM55-negative/C3-negative group (n = 22); KM55-negative/C3-positive group (n = 10); KM55-positive/C3-negative group (n = 6); and KM55-positive/C3-positive group (n = 8). The immunostaining characteristics in the subgroups are shown in Fig 1.

Urine and blood samples were collected from the recipients before puncture on the day of allograft biopsy. After centrifugation, the serum and urine supernatants were frozen at −80°C until measurements were taken. Serum and urinary Gd-IgA1 levels were measured using enzyme-linked immunosorbent assay kits (#27600; Immuno-Biological Laboratories) [18, 20].

Kidney function in the recipients was determined based on the estimated glomerular filtration rate (eGFR) calculated using the Modification of Diet in Renal Disease Study equation modified for the Japanese population [35]. The IgA/C3 ratio was calculated by dividing the serum IgA level by the serum C3 level. Urinary protein was evaluated using the urinary protein/creatinine ratio (g/gCr); a value of ≥0.15 g/gCr was considered positive for proteinuria. Urine was examined for red blood cells, and a finding of ≥5 cells/high power field was defined as positive for hematuria.

Basiliximab was used for induction therapy in all cases. Maintenance immunosuppressive regimens were based principally on tacrolimus/cyclosporine, mycophenolate mofetil, and methylprednisolone, which were used in combination.

Forty-six recipients with IgA deposition were categorized according to the original disease as those with a confirmed diagnosis of IgA nephropathy in the native kidney biopsy (recurrence group, n = 14), those with a confirmed diagnosis of non-IgA nephropathy (de novo group, n = 8), and those with no native kidney biopsy (unknown group, n = 22). Fifteen of these 46 recipients underwent subsequent allograft biopsy during the 3 years after enrollment. These 15 recipients were divided into those in whom the subsequent allograft kidney biopsy showed disappearance of IgA deposits (n = 10) and those in whom IgA deposits remained (n = 5).

### Statistical analysis

Normally distributed variables are expressed as the mean and standard deviation and non-normally distributed variables as the median and interquartile range. The Kolmogorov–Smirnov test was used to assess the normality of distributions. Categorical variables were compared between groups using the chi-squared test. Continuous variables were compared between groups using the Mann–Whitney test or the Kruskal–Wallis nonparametric test with Dunn’s multiple comparison post-hoc correction. All analyses were performed using SPSS for Windows software (version 26.0; IBM Japan, Tokyo, Japan). A *p*-value of <0.05 was considered statistically significant.

## Results

### Background characteristics of recipients

The background characteristics are shown in Table 1. More than 80% of cases had protocol biopsies without abnormal urinalysis. The median time since transplantation was 18 months, and the median eGFR was 47 mL/min/1.73 m^2^. Nine of the 28 recipients in the group with IgAN as the original disease had undergone tonsillectomy before transplantation.

**Table 1:** Background characteristics of recipients.

Recipients, n	106
Living donor kidney transplantation, n (%)	6 (6)
Male, n (%)	69 (67)
Age at biopsy, years	49.3 ± 13.6
Episode biopsy, n (%)	17 (16)
IgAN as original disease, n	28 (27)
Preemptive kidney transplantation, n	60 (58)
eGFR, mL/min/1.73 m^2^	46.7 [37.6, 53.5]
Urinary protein, g/gCr	0.13 [0.06, 0.49]
U-RBC >5/HPF, n (%)	22 (21)
Time from transplant, months	17.5 [12.1, 52.8]

Data are shown as the mean ± standard deviation, number (percentage), or median [interquartile range].

eGFR, estimated glomerular filtration rate; gCr, grams of creatinine; HPF, high power field; IgA, immunoglobulin A; IgAN, IgA nephropathy; U-RBC, red blood cells in urine.

Background characteristics and pathological features are shown according to subgroup in Tables 2 and 3. The C3 deposition rate tended to be higher in the KM55-positive group than in the KM55-negative group (57% vs 31%, *p* = 0.11). There was no significant difference in the duration of dialysis, frequency of IgAN as the original disease, or IgA deposition status in the donor kidney between the five groups. A history of tonsillectomy prior to kidney transplantation was more common in the IgA-positive group, but there was no significant association with C3 or KM55 deposition status. Recipients in the KM55-positive group tended to be more likely to not be taking prednisolone; however, this finding was not statistically significant.

**Table 2:** Background characteristics of recipients in the subgroups.

	IgA(-)	KM55(-)	KM55(-)	KM55(+)	KM55(+)	*p*-value
		C3(-)	C3(+)	C3(-)	C3(+)	
Recipients, n	60	22	10	6	8	
Age at biopsy, years	48.0 (12.7)	50.0 (15.4)	51.3 (13.8)	53.7 (14.5)	51.5 (16.7)	0.84
Male, n	38 (63%)	16 (72%)	9 (90%)	1 (17%)	5 (63%)	0.05*
Body mass index^a^	22.7 (4.1)	23.5 (4.6)	23.3 (5.0)	23.5 (5.6)	22.5 (4.3)	0.97
Dialysis duration, months	6.5 [0, 25]	2 [0, 30]	8 [2, 19]	5 [0, 8]	27 [2, 52]	0.52
Living donor kidney transplantation, n	56 (93%)	21 (95%)	9 (90%)	6 (100%)	8 (100%)	0.85
Preemptive kidney transplantation, n	21 (35%)	10 (45%)	2 (20%)	2 (33%)	2 (25%)	0.66
IgAN as original disease, n	15 (25%)	4 (18%)	2 (20%)	4 (67%)	3 (38%)	0.42
SBP, mmHg	128 (12)	137 (19)	127 (18)	129 (11)	127 (17)	0.38
ABO incompatible, n	10 (17%)	5 (23%)	3 (30%)	2 (33%)	2 (25%)	0.76
Donor kidney with IgA deposition, n	6 (10%)	2 (9%)	2 (20%)	0	0	0.59
Tonsillectomy before transplantation, n	2 (3%)	2 (9%)	1 (10%)	3 (50%)	1 (13%)	<0.01*
Tac/CyA, n	52/8	17/5	9/1	5/1	7/1	0.84
MMF use, n	48 (80%)	15 (68%)	10 (100%)	5 (83%)	7 (88%)	0.31
EVL use, n	15 (25%)	7 (32%)	1 (10%)	3 (50%)	0	0.17
PSL use, n	57 (95%)	22 (100%)	10 (100%)	6 (85%)	6 (75%)	0.07
eGFR, mL/min/1.73 m^2^	47.2 [40.5, 54.4]	44.3 [36.2, 50.8]	51.6 [35.8, 71.0]	42.9 [33.3, 57.2]	36.6 [29.3, 50.6]	0.34
IgA, mg/dL	186 (84)	217 (111)	221 (88)	173 (117)	282 (102)	0.08
IgG, mg/dL	954 (262)	980 (315)	920 (141)	707 (202)	1155 (315)	0.07
C3, mg/dL	97 (19)	98 (18)	94 (17)	106 (12)	89 (15)	0.46
IgA/C3 ratio	1.95 (0.87)	2.24 (1.10)	2.24 (0.62)	1.22 (0.61)	3.13 (0.85)	0.01*
UP, g/gCr	0.13 [0.05, 0.43]	0.12 [0.06, 0.37]	0.34 [0.11, 0.86]	0.18 [0.02, 0.53]	0.85 [0.01, 1.48]	0.37
UP >0.15g/gCr, n	24 (40%)	7 (32%)	6 (60%)	4 (67%)	6 (75%)	0.06
U-RBC >5/HPF, n	13 (22%)	2 (9%)	4 (40%)	1 (17%)	2 (25%)	0.37

The data are shown as the mean (standard deviation), number (percentage), or median [interquartile range]. ^a^Calculated as kg/m^2^. *p<0.05 for Kruskal–Wallis nonparametric test or chi-squared test between five groups; ^†^*p*<0.05 vs KM55(+)C3(−) group, ^‡^*p*<0.05 vs. IgA(−) group by Dunn’s multiple comparison post-hoc correction.

IgA, immunoglobulin A; IgA(−), IgA-negative group; IgA(+), IgA-positive group; KM55(−), KM55-negative group; KM55(+), KM55-positive group; KM55(−)C3(−), KM55-negative/C3-negative group; KM55(−)C3(+), KM55-negative/C3-positive group; KM55(+)C3(−), KM55-positive/C3-negative group; KM55(+)C3(+), KM55-positive/C3-positive group. CyA, cyclosporine; eGFR, estimated glomerular filtration rate; EVL, everolimus; gCr, grams creatinine; HPF, high-power field; IgAN, IgA nephropathy; MMF, mycophenolic mofetil; PSL, prednisolone; SBP, systolic blood pressure; Tac, tacrolimus; UP, urinary protein; U-RBC, red blood cells in urine.

**Table 3:** Pathological characteristics of allograft biopsies in the subgroups.

	IgA(-)	KM55(-)	KM55(-)	KM55(+)	KM55(+)	*p*-value
		C3(-)	C3(+)	C3(-)	C3(+)	
n	60	22	10	6	8	
Time since transplant, months	18 [12, 38]	12 [12, 37]	55 [29, 60]	18 [12, 66]	47 [19, 141]	0.06
Episode biopsy, n	7 (12%)	5 (23%)	3 (30%)	0	2 (25%)	0.32
IgA staining 2+, n	N/A	4 (18%)	0	3 (50%)	5 (63%)	0.01*
Mesangial IgG deposition, n	0	0	0	0	1 (13%)	0.01*
Glomerular C4d positive, n	8	1	0	0	3	0.07
Sclerotic glomeruli, %	14 [5, 25]	0 [0, 10]	17 [6, 33]	16 [9, 26]	15 [5, 55]	0.46
Mesangial proliferation, n	1 (2%)	0	0	0	2 (25%)	<0.01*
Endocapillary proliferation, n	1 (2%)	0	0	1 (17%)	0	0.1
Crescent formation, n	0	0	0	0	0	-
T-cell-mediated acute rejection, n	2 (3%)	1 (5%)	0	0	0	0.91
Antibody-mediated rejection, n	11 (18%)	4 (18%)	1 (10%)	1 (17%)	3 (38%)	0.67

The data are shown as the mean (standard deviation), number (percentage), or median [interquartile range]. *p<0.05 for Kruskal–Wallis nonparametric test or chi-squared test between five groups: ^†^*p*<0.05 vs KM55(+)C3(−) group, ^‡^*p*<0.05 vs IgA(−) group by Dunn’s multiple comparison post-hoc correction.

IgA, immunoglobulin A; IgA(−), IgA-negative group; IgA(+), IgA-positive group; KM55(−), KM55-negative group; KM55(+), KM55-positive group; KM55(−)C3(−), KM55-negative/C3-negative group; KM55(−)C3(+), KM55-negative/C3-positive group; KM55(+)C3(−), KM55-positive/C3-negative group; KM55(+)C3(+), KM55-positive/C3-positive group. CyA, cyclosporine; eGFR, estimated glomerular filtration rate; EVL, everolimus; gCr, grams of creatinine; HPF, high-power field; IgAN, IgA nephropathy; MMF, mycophenolate mofetil; PSL, prednisolone; SBP, systolic blood pressure; Tac, tacrolimus; UP, urinary protein; U-RBC, red blood cells in urine.

There was no significant difference in eGFR between the subgroups. However, there was a trend toward a lower eGFR in the KM55-positive/C3-positive group. Serum IgG levels and the IgA/C3 ratio were higher in the KM55-positive/C3-positive group than in the groups that were C3-positive alone, KM55-positive alone, and IgA-positive alone. There was no significant difference in the proteinuria or hematuria positivity rates between the subgroups; however, proteinuria and hematuria were found in some cases in the C3-positive groups regardless of whether KM55 was positive or negative.

The duration since transplant tended to be longer in the C3-positive groups regardless of KM55 status. Light microscopy did not show mesangial proliferation or any acute lesions, such as crescent formation or endocapillary proliferation, in most of the specimens because the majority were protocol biopsies. The T-cell-mediated rejection and antibody-mediated rejection rates were similar between the five subgroups. The percentage of specimens with 2+ IgA staining was higher in the KM55-positive group. Glomerular C4d was positive in three of the eight KM55-positive/C3-positive cases.

### Serum and urinary Gd-IgA1 levels

**Fig 2:**
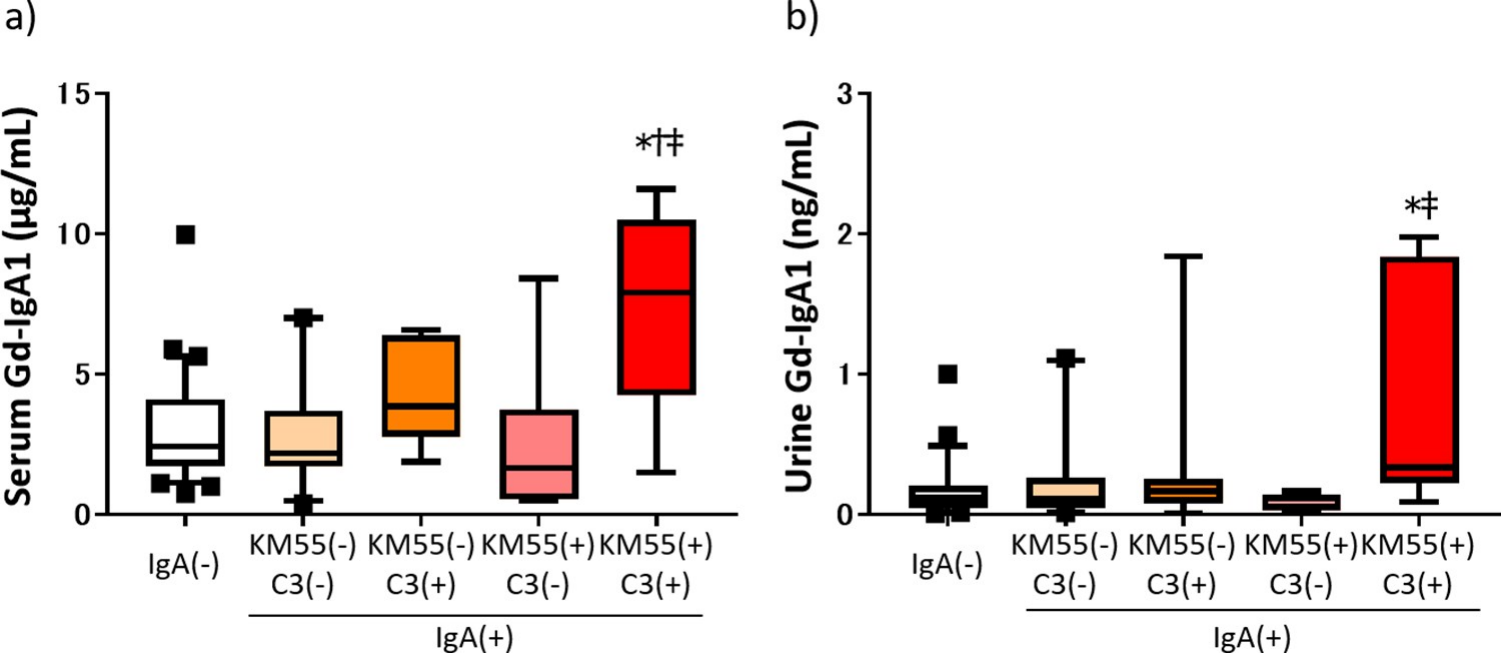
Serum and urinary Gd-IgA1 levels in the subgroups. a) Serum Gd-IgA1 levels. b) Urinary Gd-IgA1 levels. *p<0.05 vs IgA(−) group, ^†^*p*<0.05 vs. KM55(−)C3(−) group, ^‡^*p*<0.05 vs KM55(+)C3(−) group by Dunn’s multiple comparison post-hoc correction. IgA, immunoglobulin A; Gd-IgA1, galactose-deficient IgA1; IgA(−), IgA-negative group; IgA(+), IgA-positive group; KM55(−), KM55-negative group; KM55(+), KM55-positive group; KM55(−)C3(−), KM55-negative/C3-negative group; KM55(−)C3(+), KM55-negative/C3-positive group; KM55(+)C3(−), KM55-positive/C3-negative group; KM55(+)C3(+), KM55-positive/C3-positive group.

**Fig 3:**
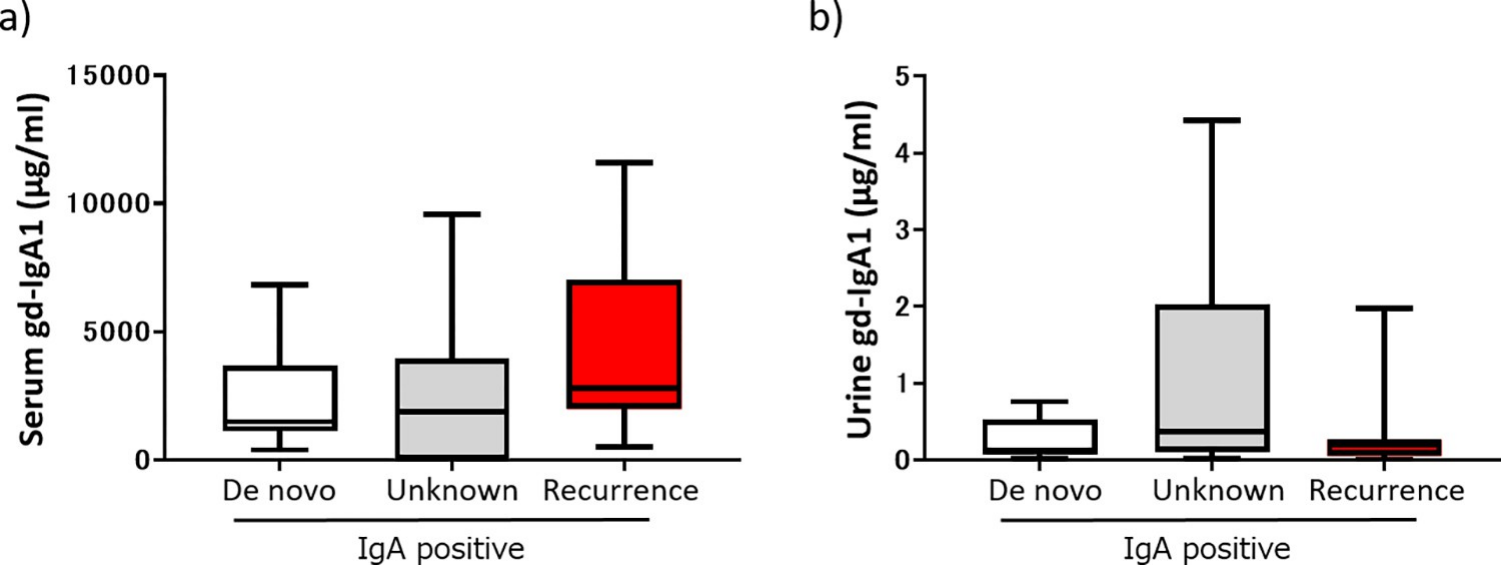
Serum and urinary Gd-IgA1 levels according to the original disease. a) Serum Gd-IgA1 levels. b) Urinary Gd-IgA1 levels. Gd-IgA1, galactose-deficient IgA1; IgA, immunoglobulin A.

Serum and urinary Gd-IgA1 levels are shown according to subgroup in Fig 2. There was no significant difference in serum Gd-IgA1 levels between the groups that were positive for IgA alone, C3 alone, or KM55 alone and the cases that were IgA-negative. Serum Gd-IgA1 levels were significantly higher in the KM55-positive/C3-positive group than in the IgA-negative group, KM55-negative/C3-negative group, and KM55-positive/C3-negative group. A similar result was observed for urinary Gd-IgA1. Serum and urinary Gd-IgA1 levels are shown according to the original disease and IgA deposition status in Fig 3. There was no significant difference in the serum or urinary Gd-IgA1 levels according to the original disease.

### Relationship between quantitative assessment of Gd-IgA1 and prognosis according to IgA deposition status

In the 15 IgA-positive recipients who had undergone subsequent allograft biopsy, IgA deposition was confirmed to be remaining in 5 and to have disappeared in 10. In all 15 cases, the indication for subsequent allograft biopsy was protocol biopsy. The clinical characteristics of these patients at the time of enrollment are compared according to whether IgA deposits remained or disappeared in Table 4. There was no significant between-group difference in eGFR, frequency of abnormal urinalysis, KM55 positivity rate at enrollment, or average period from enrollment to subsequent biopsy. None of the recipients were treated for IgA deposition after enrollment in the study; however, corticosteroid pulse therapy was administered for coexistent focal segmental glomerulosclerosis in one patient in whom IgA deposits disappeared. There was no significant between-group difference in eGFR or in urinary protein or red blood cells. There were no cases of allograft loss during the observation period in recipients for whom follow-up information was available. The 10 recipients in whom IgA deposition disappeared included five KM55-negative/C3-negative cases, one KM55-negative/C3-positive case, two KM55-positive/C3 negative cases, and two KM55-positive/C3-positive cases. The five patients in whom IgA deposition remained included two KM55-negative/C3-negative cases and three KM55-positive/C3-positive cases. In the subsequent biopsy, the deposition of IgA and C3 was consistent; in the disappeared group, C3 deposition in the subsequent allograft biopsy was negative in all 10 cases (3 were C3-positive at the time of enrollment). In the remaining group, C3 deposition in the subsequent allograft biopsy was positive in all five cases (three were C3-negative at the time of enrollment).

**Table 4:** Characteristics of recipients in the group in which IgA deposits disappeared and in the group in which they remained.

	Group with disappearance of IgA	Group with remaining IgA	*p*-value
Recipients, n	10	5	
Age at biopsy, year	47 (18)	49 (15)	0.67
Period from enrollment to subsequent biopsy, month	50 [8–53]	22 [7–60]	0.18
IgAN as original disease, n	2 (20%)	1 (20%)	0.99
eGFR at biopsy, mL/min/1.73 m^2^	49.8 [35.7, 55.8]	43.7 [36.6, 51.6]	0.65
⊿eGFR, mL/min/1.73 m^2^	−2.6 (12.7)	−3.6 (4.9)	0.31
UP at biopsy, g/gCr	0.14 [0.05, 1.08]	0.20 [0.03, 0.97]	0.75
⊿UP, g/gCr	0 [−0.53, 0.03]	0 [-0.25, 2.8]	0.38
U-RBC >5/HPF at baseline, n	2 (20%)	2 (40%)	0.41
U-RBC >5/HPF at re-biopsy, n	2 (20%)	1 (20%)	0.99
KM55-positive, n	4 (40%)	3 (60%)	0.46
Mesangial C3-positive, n	3 (30%)	3 (60%)	0.26
Tonsillectomy after enrollment	0	0	-
Prednisolone pulse therapy after inclusion	1 (10%)	0	1.0
ARB use after enrollment	1 (10%)	0	1.0

Data are shown as the mean (standard deviation) or as the median [interquartile range] or as the number (percentage).

*p<0.05 by Mann–Whitney test or chi-squared test between group.

ARB, angiotensin receptor blocker; eGFR, estimated glomerular filtration rate; gCr, grams of creatinine; HPF, high-power field; IgAN, immunoglobulin A nephropathy; UP, urinary protein; U-RBC, red blood cells in urine.

**Fig 4:**
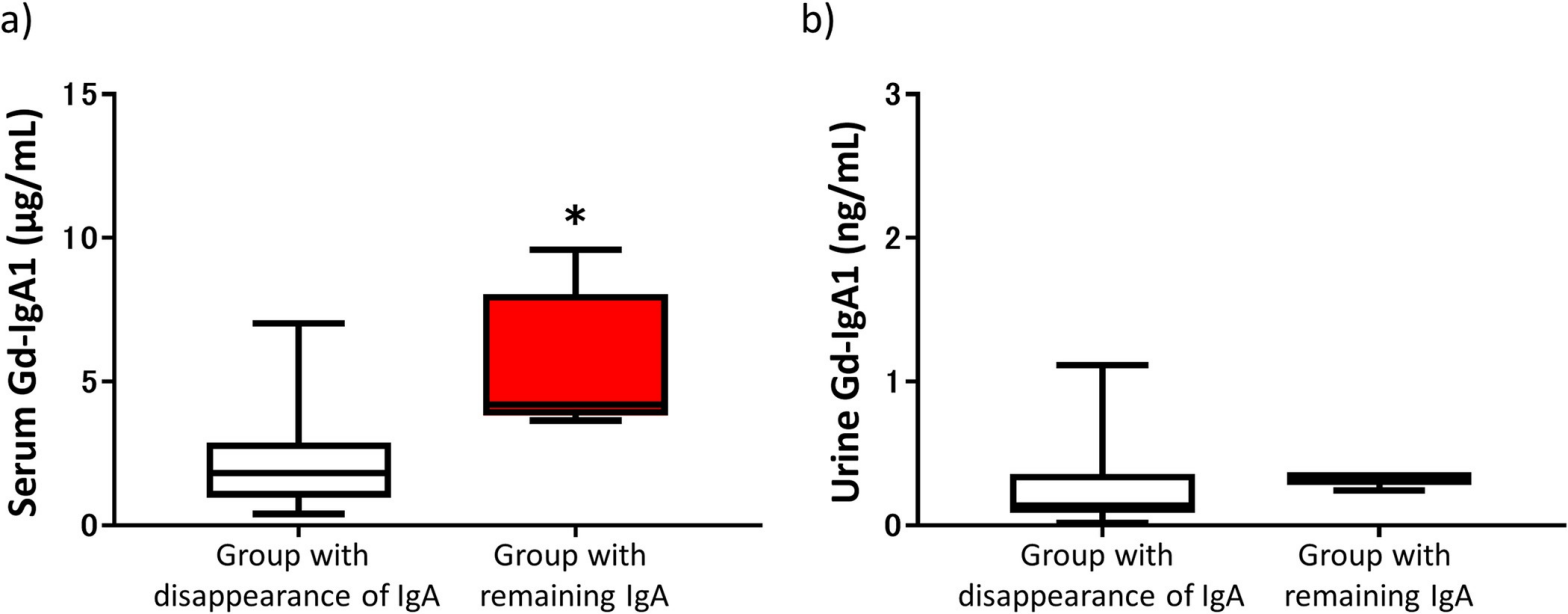
Serum and urinary Gd-IgA1 levels at the time of enrollment in the group in which IgA deposits disappeared and the group in which it remained. a) Serum Gd-IgA1 levels. b) Urinary Gd-IgA1 levels. Gd-IgA1, galactose-deficient IgA1; IgA, immunoglobulin A. *p<0.05 vs group with disappearance of IgA.

Serum and urinary Gd-IgA1 levels at the time of enrollment are shown according to whether IgA deposition remained or disappeared in Fig 4. Serum Gd-IgA1 levels at enrollment were significantly higher in the group in which IgA deposition remained than in the group in which it disappeared (*p* = 0.02). However, there was no significant between-group difference in urinary Gd-IgA1 levels.

## Discussion

In this study, we found that IgA deposition in renal allografts was serologically and pathologically heterogeneous. Moreover, serum and urinary Gd-IgA1 levels were higher in recipients who were both KM55-positive and C3-positive than in those who were positive for IgA alone, KM55 alone, or C3 alone. In the longitudinal analysis, patients with IgA deposition that remained had higher serum Gd-IgA1 levels at the time of enrollment. These findings suggest that serological and pathological evaluation of Gd-IgA1 may be useful for predicting disease activity and prognosis of post-transplant IgA deposition.

The present study is the first report to analyze both the serological and pathological characteristics of Gd-IgA1 in renal allografts with IgA deposition. In our recipients, these characteristics appeared to be homogeneous on conventional IgA staining and total serum IgA quantification but showed heterogeneity on serological and pathological Gd-IgA1 assessment. We previously reported that KM55 staining was positive in allografts with IgA deposition [24]. In this cohort, 30% of transplant recipients with IgA deposition had histological evidence of Gd-IgA1 deposition. Elevated Gd-IgA1 levels have been reported in patients with IgAN in their allograft as well as in their native kidney [18, 36]. Differences in the quality of abnormalities in IgA glycosylation between recipients with IgAN and donors with IgA deposition have also been reported [37]. In our cohort, cases with histologically co-positive KM55 and C3 had serologically elevated gd-IgA1. After evaluation of the long-term prognosis, it could be possible to classify post-transplant IgA deposition based on serological and histological evaluation of Gd-IgA1.

In this study, transplant recipients who were both KM55-positive and C3-positive had more post-transplant years, higher rates of positivity for proteinuria and hematuria, and high serum and urinary Gd-IgA1 levels. This group of patients may have included cases in which IgA deposition recurred asymptomatically and then progressed to symptomatic IgAN. Recipients who were positive for only KM55 were more likely to have a relatively short post-transplant period and may have been in the process of developing recurrence. Whether recipients in these populations would progress to being positive for both KM55 and C3 if they have high Gd-IgA1 levels could not be determined during our study period. However, serum Gd-IgA1 levels were low in recipients with IgA deposition if they were not KM55-positive or C3-positive, suggesting that these patients had low disease activity.

The pathogenesis of IgAN can be observed in allografts [16]. In the present study, we could not detect IgA deposition in allografts using biomarkers, which might reflect in part the fact that the total serum IgA level is reduced by immunosuppressive agents [38]. Moreover, we could not compare the serological status of Gd-IgA1 in the recipients with that in native kidneys because asymptomatic IgA deposition cannot be detected in the native kidney. Furthermore, IgA deposition in an allograft is less likely to be associated with abnormal findings on urinalysis and might not be significant enough to be detected non-invasively. However, evaluation of serum and urinary Gd-IgA1 levels might allow assessment of the need for biopsy of a kidney allograft in centers that do not perform protocol biopsy.

IgA deposition in donated kidneys almost disappears within 1 year after transplantation [9, 12, 39]. In this study, IgA deposition disappeared in 66% of recipients who underwent a subsequent allograft biopsy, which is consistent with reports of latent IgA deposition in donated kidneys. Therefore, we believe that the IgA deposition observed in this study reflects recurrent or de novo deposition of IgA rather than remaining IgA deposits in donated kidneys. Our findings also demonstrate that asymptomatic IgA deposition detected by protocol biopsy is associated with a favorable prognosis. Furthermore, IgA deposition was observed to disappear in both KM55-positive and C3-positive cases. Although the mechanism of disappearance of IgA from the mesangial area has not been elucidated in detail, our recipients in whom IgA deposits disappeared were found to have lower serum Gd-IgA1 levels at the time of enrollment. Given that 80% of cases were confirmed by electron microscopy to have electron-dense deposits, the possibility of false positives due to technical problems can be ruled out. If the serum Gd-IgA1 level can predict the disappearance of IgA deposition, unnecessary treatment might be avoided.

Whether or not IgAN is the original cause of end-stage kidney disease is a factor that influences the treatment strategy for post-transplant IgA deposition. When recurrent IgA deposition is observed in recipients, tonsillectomy and/or corticosteroid pulse therapy are often performed [25, 40–42]. However, when IgA deposition is observed and the original renal disease is unknown, it is impossible to determine whether IgA deposition is recurrent or de novo. Given the difficulties in accurate diagnosis of the original disease prior to kidney transplantation, it is difficult to determine the indication for treatment based on the original disease alone. It is assumed that disease activity is not high in cases of asymptomatic IgA deposition that are de novo with low serum Gd-IgA1 levels. Our results suggest that specific treatment should be limited to recipients with clinical recurrence, high serum Gd-IgA1 levels, and/or co-deposition of C3 with abnormal urinary findings.

This study has some limitations. First, the small number of recipients in the IgA subgroup made the design of the study descriptive and preliminarily. Second, the observation period after detection of IgA deposition was relatively short. Therefore, we are unable to evaluate the risk of graft loss in the long term analysis. Third, biomarker sampling and KM55 staining at the time of subsequent allograft biopsy were not available in this study. Evaluation of these assessments should be made in future prospective cohorts. Finally, although an association between anti-Gd-IgA1-specific autoantibodies and IgA deposition in the allograft has been reported [28], anti-Gd-IgA1-specific autoantibodies were not investigated in this study. However, other researchers found that serum anti-Gd-IgA1-specific autoantibodies did not predict outcome in the native kidney [36]. Nevertheless, the multicenter prospective design of our study eliminates uncertainties arising from regional differences and differences in the regimens used.

In conclusion, the population with IgA deposition after kidney transplantation is serologically and pathologically heterogeneous. Our findings indicate that serological and histological assessment of Gd-IgA1 is useful for identifying cases that should be carefully observed.

## Supporting information

S1 Fig Flow chart summarizing the patient classification process. IgA, immunoglobulin A; IgA(−), IgA-negative group; IgA(+), IgA-positive group; KM55(−), KM55-negative group; KM55(+), KM55-positive group; KM55(−)C3(−), KM55-negative/C3-negative group; KM55(−)C3(+), KM55-negative/C3-positive group; KM55(+)C3(−), KM55-positive/C3-negative group; KM55(+)C3(+), KM55-positive C3-positive group.
(TIF)

S1 Table Background characteristics of recipients in the IgA-negative original cohort and the IgA-positive extracted cohort. (PDF)
