# Supplementary figures and images for: Serological and histopathological assessment of galactose-deficient immunoglobulin A1 deposition in kidney allografts: A multicenter prospective observational study
Source: PLoS One. 2023 Feb 16;18(2):e0281945. doi: 10.1371/journal.pone.0281945 (PMC9934455; doi:10.1371/journal.pone.0281945)

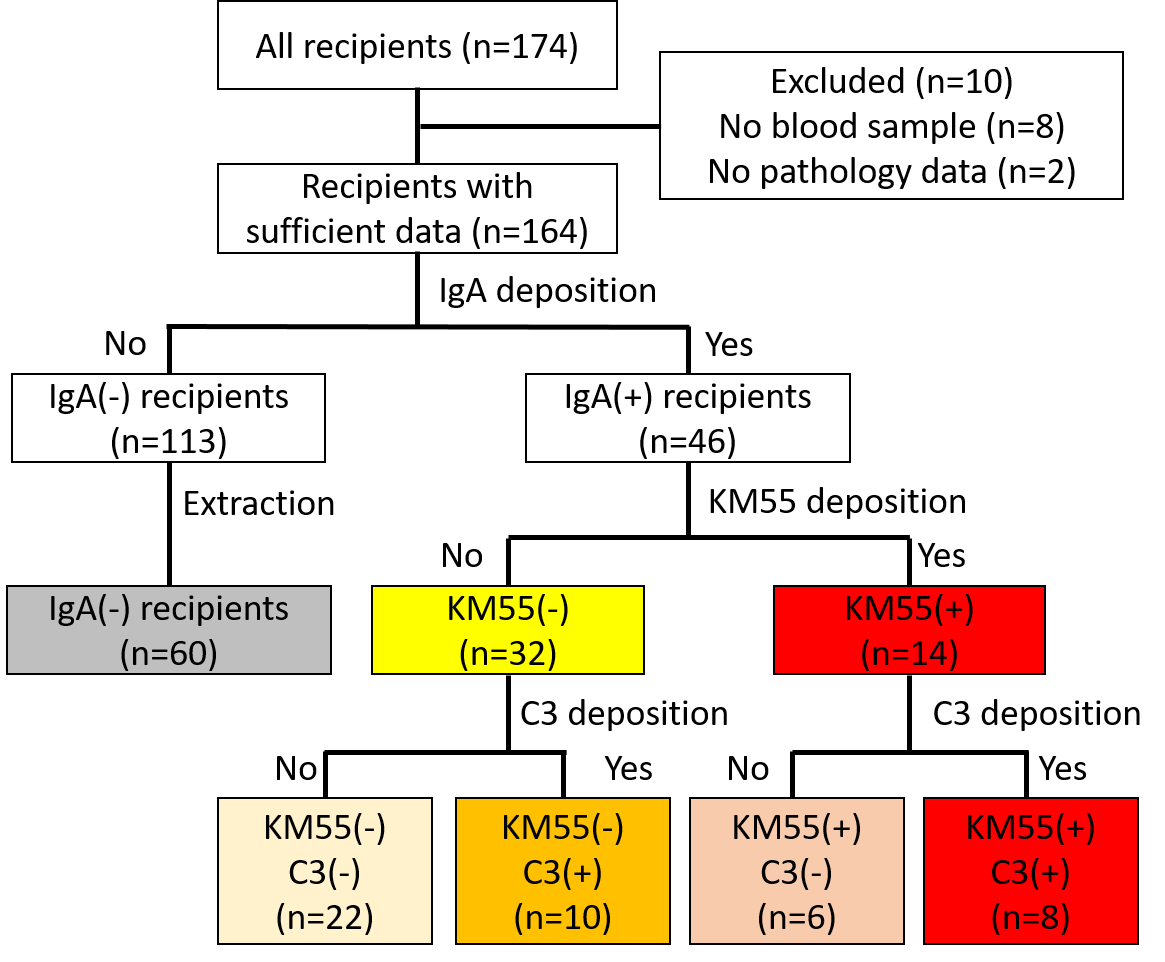

Supplement: S1 Fig — IgA, immunoglobulin A; IgA(−), IgA-negative group; IgA(+), IgA-positive group; KM55(−), KM55-negative group; KM55(+), KM55-positive group; KM55(−)C3(−), KM55-negative/C3-negative group; KM55(−)C3(+), KM55-negative/C3-positive group; KM55(+)C3(−), KM55-positive/C3-negative group; KM55(+)C3(+), KM55-positive C3-positive group. (TIF) [file pone.0281945.s001.tif]
