# Supplementary material for: Serological and histopathological assessment of galactose-deficient immunoglobulin A1 deposition in kidney allografts: A multicenter prospective observational study
Source: PLoS One. 2023 Feb 16;18(2):e0281945. doi: 10.1371/journal.pone.0281945 (PMC9934455; doi:10.1371/journal.pone.0281945)
Supplement: S1 Table — (PDF) [file pone.0281945.s002.pdf]

S1 Table.

	**IgA-negative original cohort**	**IgA-negative extracted cohort**	***p*-value**
Recipients, n	113	60	
Age at biopsy, years	50.4 (13.9)	48.0 (12.7)	0.27
Male, n	71 (63%)	38 (63%)	0.95
Body mass index[a]	22.3 (4.0)	22.7 (4.1)	0.54
Dialysis duration, months	8 [0, 27]	6.5 [0, 25]	0.59
PEKT, n	44 (39%)	21 (35%)	0.63
IgAN as original disease, n	24 (21%)	15 (25%)	0.57
SBP, mmHg	126 (14)	128 (12)	0.52
ABO incompatible, n	22 (20%)	10 (17%)	0.99
Donor kidney with IgA deposition, n	9 (8%)	6 (10%)	0.78
Tonsillectomy before transplantation, n	6 (5%)	2 (3%)	0.72
Tac use, n	94 (83%)	52 (87%)	0.66
MMF use, n	92 (81%)	48 (80%)	0.84
EVL use, n	25 (22%)	15 (25%)	0.71
PSL use, n	108 (96%)	57 (95%)	0.99
eGFR, mL/min/1.73m^2	47.6 [39.5, 55.0]	47.3 [40.5, 54.4]	0.77
IgA, mg/dL	187 (94)	186 (84)	0.97
IgG, mg/dL	947 (260)	954 (262)	0.87
C3, mg/dL	96 (18)	97 (19)	0.79
IgA/C3 ratio	1.98 (1.14)	1.95 (0.87)	0.84
UP, g/gCr	0.11 [0.04, 0.39]	0.13 [0.05, 0.43]	0.56
Time since transplant, months	16.4 [12.1, 61.4]	17.5 [12, 38]	0.3
Episode biopsy, n	19 (17%)	7 (12%)	0.5
Mesangial C3 deposition, n	6 (5%)	2 (3%)	0.72

The data are shown as the mean (standard deviation) or as the median [interquartile range]. [a]Calculated as kg/m^2. *$p<0.05$. CyA, cyclosporine; eGFR, estimated glomerular filtration rate; EVL, everolimus; gCr, grams

creatinine; IgAN, immunoglobulin A nephropathy; MMF, mycophenolate mofetil; MP, methylprednisolone; PEKT, preemptive kidney transplantation; SBP, systolic blood pressure; Tac, tacrolimus; UP, urinary protein; U-RBC, red blood cells in urine
